# Controlled Delivery and Light‐Induced Release of Magic Spot Nucleotides in *Escherichia coli*

**DOI:** 10.1002/anie.202523307

**Published:** 2026-02-25

**Authors:** Christoph Popp, Patrick Moser, Anselm Schwoerbel, Xinwei Liu, Johannes Freitag, Pinku Sarmah, Isabel Prucker, Robert Zscherp, Xuan Wang, Philipp Klahn, Hans‐Georg Koch, Gert Bange, Henning J. Jessen

**Affiliations:** ^1^ Faculty of Chemistry and Pharmacy Institute of Organic Chemistry & CIBSS Centre For Integrative Biological Signalling Studies Albert‐Ludwigs University Freiburg Freiburg Germany; ^2^ Center For Synthetic Microbiology (SYNMIKRO) Marburg University Marburg Germany; ^3^ Faculty of Medicine Institute For Biochemistry and Molecular Biology, ZBMZ Albert‐Ludwigs University Freiburg Freiburg Germany; ^4^ Department of Chemistry and Molecular Biology Division of Organic and Medicinal Chemistry University of Gothenburg Gothenburg Sweden

**Keywords:** Cellular delivery, magic spot nucleotides, photocages, ppGpp, stringent response

## Abstract

The “magic spot” nucleotides (MSNs) ppGpp and pppGpp constitute bacterial alarmones that orchestrate the conserved stringent response, a global regulatory mechanism enabling bacteria to adapt to nutrient deprivation and other environmental stresses. Current strategies to manipulate MSN levels rely mainly on genetic or environmental approaches, which are slow and lack temporal control. Chemical tools such as photocaged MSN analogues could provide such temporal control over MSN levels. However, the high negative charge of MSNs prevents spontaneous passage through the complex bacterial cell envelope. Here, we report the synthesis of photocaged, clickable, and isotope‐labeled MSN analogues and their delivery into *Escherichia coli* comparing different approaches. A cyclodextrin‐based synthetic nucleotide transporter facilitated uptake. Upon 400 nm irradiation, these probes were photo‐released inside living cells, where we tracked their conversion from pppGpp to ppGpp by capillary electrophoresis mass spectrometry and demonstrated their ability to alter growth in a (p)ppGpp^0^ mutant. These probes lay the foundation for spatially and temporally controlled studies of MSN function and of other highly negatively charged metabolites in vivo.

## Graphical abstract

We synthesized photocaged, clickable and isotope‐labeled “magic spot” Nucleotides and delivered them into *Escherichia coli* comparing different approaches. Light‐controlled release inside living cells enabled tracking of their conversion from pppGpp to ppGpp by capillary electrophoresis mass spectrometry and revealed their impact on the growth rate of a (p)ppGpp^0^ mutant.

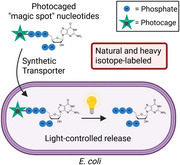

## 1 Introduction

**FIGURE 1:**
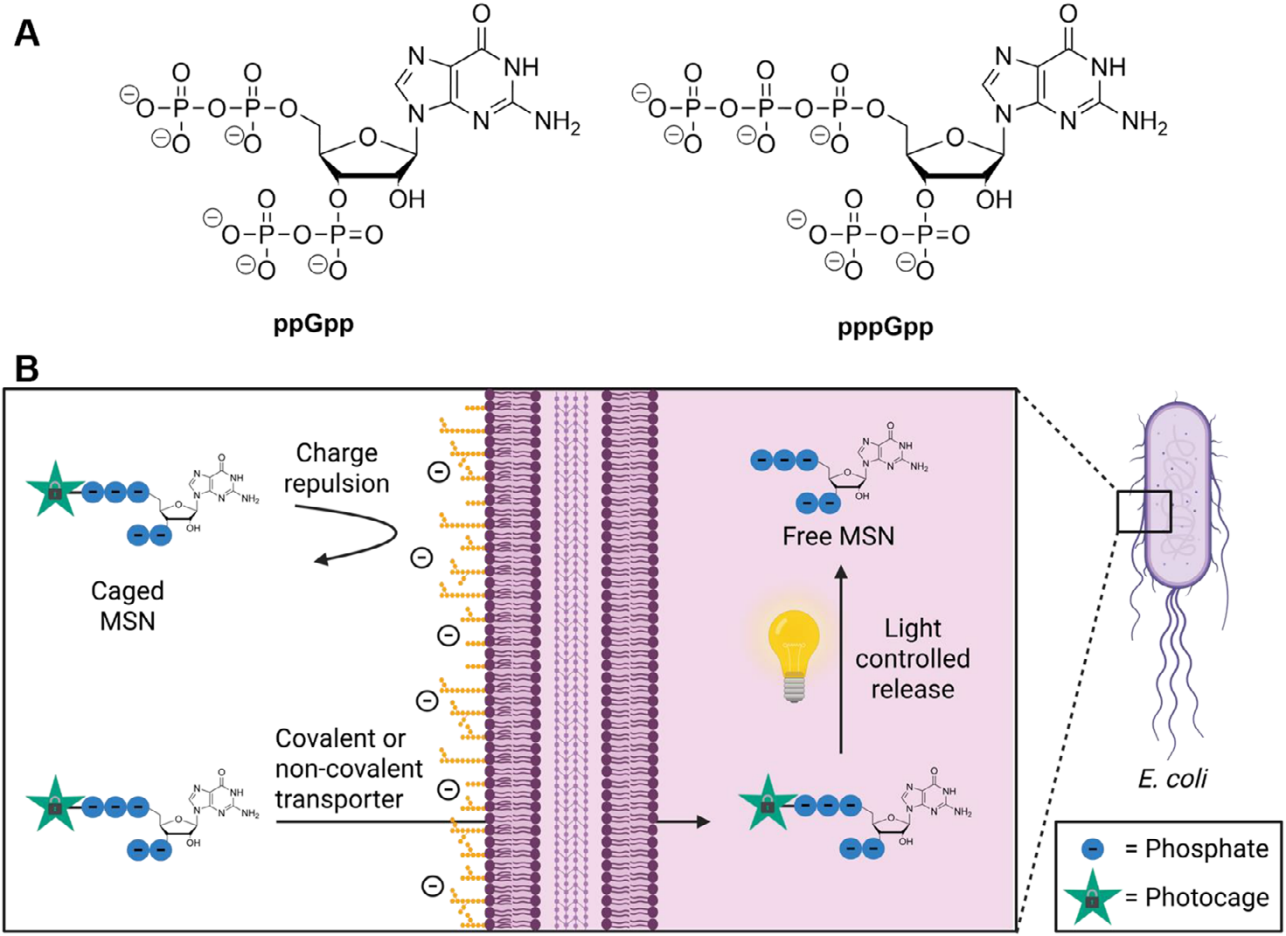
(A) Chemical structure of ppGpp and pppGpp. (B) Overview of our strategy to modulate MSN concentrations inside living cells. Due to charge repulsion from the negatively charged lipopolysaccharides and lipids, MSNs cannot readily pass through the complex bacterial cell envelope. We tested various strategies to deliver a photocaged MSN analogue into the bacterial cell and release the MSNs by light irradiation. Created in BioRender. Popp, C. (2026) https://BioRender.com/wnqejr2.

When bacteria are exposed to stress conditions like temperature change, pH change, starvation, or antibiotics, alarmones such as guanosine tetraphosphate (ppGpp) and guanosine pentaphosphate (pppGpp) accumulate and induce the stringent response (Figure 1A) [1, 2, 3, 4, 5]. Due to their “magic” appearance on autoradiograms of amino acid starved *Escherichia coli* extracts, these nucleotides are also referred to as magic spot nucleotides (MSNs) [5]. MSNs help bacteria to survive by inducing a dormant‐like state until conditions improve. The extent of stringency is a function of MSN concentration. Beyond stress adaptation, MSNs also act as second messengers during normal growth, where basal levels are essential for regulating transcription, translation, replication, and metabolism through a wide network of protein interactions [4, 6].

Approaches to manipulate the abundance of MSNs in cellular environment include genetic modifications, treatment with small molecule drugs or exposure to stress conditions [7, 8, 9]. It would be highly desirable to study effects of local, constrained pools of these molecules. Genetic modification is time consuming and often causes secondary effects. In addition, MSN‐producing enzymes can synthesize both pppGpp and ppGpp, making it difficult to address the response to a particular MSN. To influence concentration levels of single MSNs further genetic modifications are needed [10]. Thus, an alternative chemical biology approach for modulation of MSN levels inside bacteria would provide new avenues for complementary experiments and enable further investigations into the effects of MSNs on bacterial physiology. The aim of this study was the delivery of photocaged MSNs into bacterial cells, thus enabling their controlled release by light (Figure 1B). Since in vitro data of *E. coli* and *Bacillus subtilis* MSN synthesizing enzymes RelA and SAS1 show that MSNs can stimulate their own synthesis, the introduction of small amounts of MSNs into the bacteria might be sufficient to induce higher cellular concentrations. [8, 11, 12, 13, 14, 15]

The main obstacle for this approach is the bacterial cell envelope. In contrast to the lipid bilayer of mammalian cells, bacteria possess a much more challenging barrier. In Gram‐negative bacteria, the cell wall is composed of several peptidoglycan layers surrounded by an outer membrane containing phospholipids and lipopolysaccharides. MSNs can carry up to seven negative charges that impair translocation through the negatively charged cell envelope [16, 17]. Several methods have been developed to transport highly negatively charged small molecules like nucleotides into mammalian cells. For example, prodrugs with masked negative charges and/or lipophilic groups [18, 19, 20, 21], positively charged polymers [22, 23], covalently linked cationic groups [24, 25], permeabilization of the plasma membrane with surfactants [26] or by physical and mechanical means [27, 28, 29, 30]. In bacteria, however, delivery studies of charged metabolites are very limited and only few examples are known. It is possible to transport non‐natural nucleotides into bacterial cells by heterologous expression of importers from other species [31, 32]. However, this approach is limited to several non‐natural nucleotides and requires genetic modification. Carlson and co‐workers reported the development of a cationic polymer, promoting internalization of an adenosine triphosphate derived chemical probe into *E. coli* and *B. subtilis* [33]. They proposed that the cationic polymers act as general permeabilization reagents that promote the entry of various molecules into the cytosol. Kraus and coworkers used a cyclodextrin based synthetic nucleotide transporter (SNT) [34]. It consists of a per‐6‐amino‐β‐cyclodextrin coupled to a peptide comprising eight aminocaproic acid/arginine pairs. The cyclodextrin binds the nucleotide while the peptide disrupts the membrane to facilitate cellular delivery. They suggested that the bound nucleotide is released in the cytosol due to replacement by endogenous nucleotides. Successful delivery of a fluorescently modified deoxyuridine triphosphate into live *Mycobacterium smegmatis* and *E. coli* was shown with this approach, yet the study was mainly focused on delivery into mammalian cells [34].

Other highly negatively charged molecules that are routinely transported into bacterial cells are nucleic acids. Transformation is usually done by electroporation or by heat shock of chemically competent cells [35, 36, 37, 38]. Furthermore, it is possible to use other methods such as cationic polymers and peptides [39, 40]. For mammalian cells several advanced methods have been developed, allowing targeted delivery of nucleic acids in vivo [41]. Oligonucleotides are commonly modified with phosphorotioates, making them nuclease resistant, more hydrophobic and enabling cellular uptake [42]. It should be noted, however, that for many applications involving nucleic acid delivery, the delivery of only a few copies is sufficient as these molecules can be amplified or exhibit catalytic activity. In contrast, nucleotide transport typically requires high concentrations.

For less polar or cationic substances several further methods have been developed to facilitate delivery [43, 44]. One example that showed promising results for cargo delivery into various Gram‐negative bacteria are siderophores [45, 46]. Coupling these Fe(III)‐chelating molecules to a cargo allows hijacking of the siderophore‐mediated iron uptake pathway. This approach was extensively used for antibiotic conjugates but as well to deliver larger molecules like nucleic acid therapeutics into bacteria [46, 47, 48, 49, 50, 51]. Carbohydrate conjugates were used to transport imaging probes into bacteria through the bacteria‐specific maltodextrin transport pathway [52, 53]. Additional approaches uses chalcogenides to increase cellular uptake [54], and recent studies demonstrated that disulfide‐containing conjugates increase the antibacterial activity of fidaxomicin, cephalosporin, and vancomycin [55, 56, 57]. Other approaches involve the use of nanoparticles [41, 58], cell‐penetrating peptides [59, 60, 61], or boron clusters [62].

An established method to control the release of biologically active molecules inside cells or tissue is the introduction of photolabile protecting groups, so‐called photocages [63, 64, 65, 66, 67]. These structures are covalently bound to the bioactive compound rendering them biologically inert. They can be cleaved by light irradiation. The use of light allows temporal and spatial control over the release of the biomolecule. One commonly used caging group is based on the coumarin scaffold. The coumarin photocage stands out through high biocompatibility, easy synthesis, flexibility of structural modifications, and a well‐studied mechanism of photocleavage [68, 69].

Here, we report that coumarin caged MSNs can be delivered into living *E. coli* cells using a modified cyclodextrin as additive. MSNs can be released on demand by irradiation with light and are biologically active.

## 2 Results and Discussion

### 2.1 Synthesis of Caged MSNs

**SCHEME 1:**
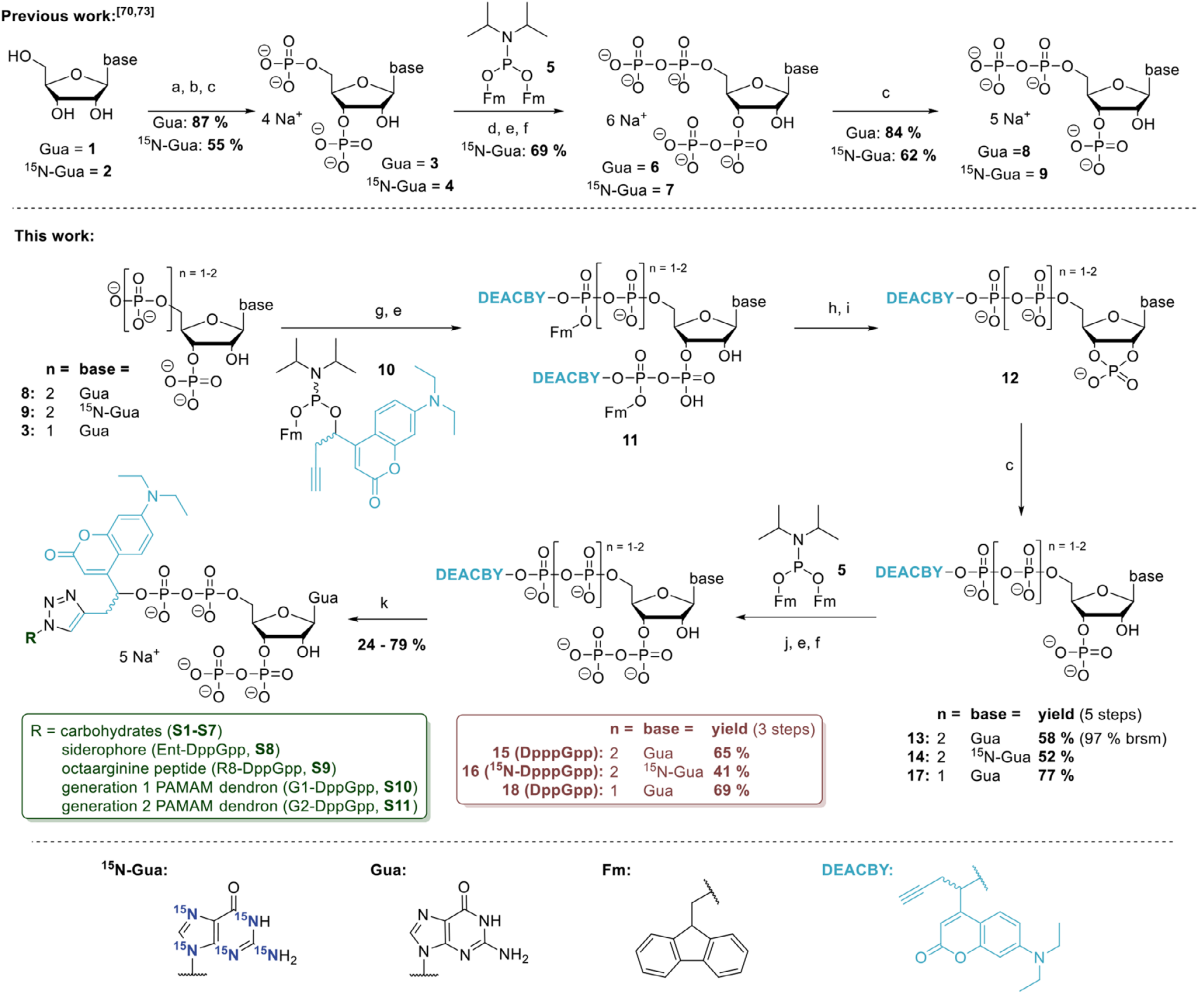
Synthesis of caged and isotope labeled MSN derivatives. [70, 73] a) P_2_Cl_4_O_3_, (11 eq), 0°C, 3 h. b) NaHCO_3_ (1 M), 0°C. c) RNase T2, 37°C, 12 h. d) 5 (3.0 eq), ETT (5.0 eq), DMF, rt, 15 min. e) *m*CPBA, –20°C, 10 min. f) DBU, 0°C→rt, 30 min. g) 10 (2.3 – 2.4 eq), ETT (5.0 eq), DMF, rt, 1 h. h) MeOH, rt, 5 min i) Piperidine, DMF, rt, 5 min. j) 5 (1.5 – 1.7 eq), ETT (3.0 – 3.5 eq), DMF, rt, 15 min. k) Azide (1.5 eq), Na‐ascorbate (5.0 eq), CuSO_4_ (0.1 eq), THPTA (0.5  eq), H_2_O, rt, 3 h. *Abbreviations*: brsm: based on recovered starting material, DBU: 1, 8‐ diazabicyclo(5.4.0)undec‐7‐ene, ETT: 5‐(ethylthio)‐1H‐tetrazole, Fm: fluorenmethyl, Gua: guanine, *m*CPBA: *meta*‐chloroperbenzoic acid, rt: room temperature. PAMAM: polyamidoamine. THPTA: tris(3‐hydroxypropyltriazolylmethyl)amine.

The synthesis of caged MSNs followed our previously reported synthetic methods employing chemoselective phosphorylation with phosphoramidites and regioselective RNase T2 hydrolysis (Scheme 1) [70, 71, 72, 73, 74]. The synthesis started with guanosine or ^15^N‐labeled guanosine, which was treated with pyrophosphoryl chloride. Controlled hydrolysis leads to the 2′‐3′ cyclophosphate, which was regioselectively opened with RNase T2 to form pGp (**3**) or ^15^N‐pGp (**4**) [70, 73]. Unlabeled and ^15^N‐labeled pGp was phosphorylated with bisfluorenmethyl phosphoramidite (**5**), oxidized and Fm‐deprotected to form ppGpp (**6**) and ^15^N‐ppGpp (**7**). ^15^N‐ppGpp was purified, while unlabeled ppGpp was used without purification. Treatment with RNase T2 and purification by strong anion‐exchange chromatography yielded ppGp (**8**) and ^15^N‐ppGp (**9**). [70, 73]

For photocaging, we adopted the 7‐(diethylamino)‐4‐(but‐3‐yn‐1‐yl)coumarin (DEACBY) chromophore introduced by Seyfried et al. [75]. We chose to use this photocage because of its easy access and further as the alkyne group would enable late‐stage functionalization of the caged MSNs by copper‐catalyzed azide‐alkyne cycloadditions to enhance uptake [76, 77].

Treatment of ppGp (**8**) and ^15^N‐ppGp (**9**) with mixed P‐amidite **10** containing the photocage DEACBY followed by oxidation with *m*CPBA led to the 3′‐diphosphate 5′‐triphosphate nucleotides with triesters at the terminal phosphates (**11**). The triester at the 3′‐end acts as a leaving group when the molecules are dissolved in MeOH allowing fast and efficient cyclization to the corresponding 2′‐3′ cyclophosphates (**12**). The fluorenmethyl (Fm) group was removed with piperidine, and the cyclophosphate was regioselectively opened with RNase T2 leading to 5′‐DEACBY protected pppGp (**13**) and ^15^N‐pppGp (**14**) in 58 % (97 % considering recovered starting material) and 52 % yield, respectively. Bisfluorenmethyl phosphoramidite reacted chemoselectively with the monoester phosphate. Oxidation and Fm deprotection with 1,8‐diazabicyclo[5.4.0]undec‐7‐en led to the caged MSNs DpppGpp (**15**) and ^15^N‐DpppGpp (16) in 65 % and 41 % yield, respectively. The efficiency of this synthesis enabled the production of 13 mg ^15^N‐labeled caged pppGpp (**16**) starting from 70 mg ^15^N‐labeled ppGpp (**7**).

To synthesize the caged magic spot nucleotide DppGpp, pGp was treated with mixed amidite **10**, oxidized, cyclized in methanol and deprotected. Regioselective ring‐opening with RNase T2 led to DppGp (**17**) in 77 % yield on a 700 mg scale. Another cycle of P‐amidite coupling with bisfluorenmethyl phosphoramidite that selectively only reacts with terminal phosphates [78], oxidation, and Fm deprotection with DBU led to the caged guanosine tetraphosphate DppGpp (**18**) in 69% yield.

The alkyne handle on the photocage now offered the possibility to easily introduce structures that could facilitate uptake into bacterial cells, while photocleavage would eventually remove them again. Various carbohydrates (glucose, galactose, mannose, maltose, lactose, trehalose), a biomimetic enterobactin analogue [48], an octaarginine cell‐penetrating peptide, and polyamidoamine (PAMAM) dendrons were readily coupled to DppGpp via copper‐catalyzed azide‐alkyne cycloadditions with yields of 24%–79% (**S1‐11**) [76, 77].

For control experiments, caged adenosine triphosphate (D‐ATP, **S12**) and caged guanosine diphosphate (D‐GDP, **S13**) were synthesized by treating commercial ADP and GMP with mixed amidite **10**. Oxidation and Fm deprotection yielded D‐ATP (**S12**) and D‐GDP (**S13**).

### 2.2 Photolysis Behavior

**FIGURE 2:**
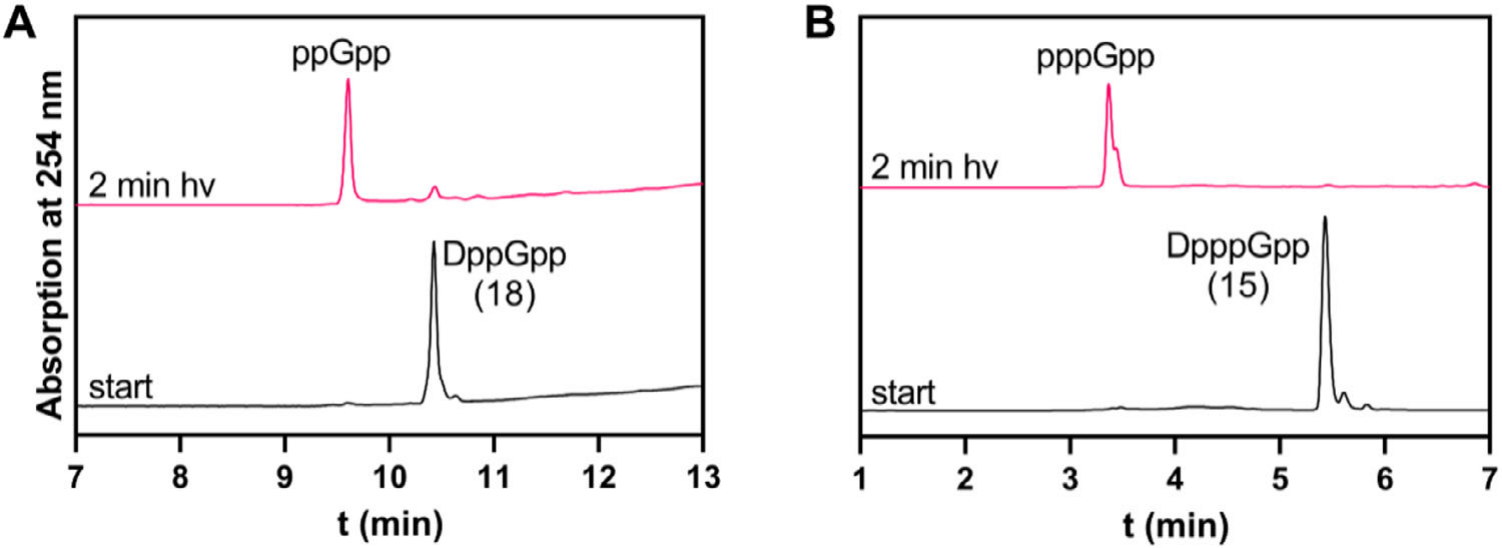
HPLC chromatograms (254 nm) of photolysis of DppGpp (18) (A) and DpppGpp (15) (B). Chromatograms were measured before and after irradiation with a 400 nm LED (25 nmol, 50 µM, 14 mW).

To study the photolysis behavior of the coumarin photocages, the caged MSNs DppGpp (**18**) and DpppGpp (15) were irradiated with a 400 nm LED in a small glass vial (25 nmol, 50 µM, 14 mW, see Figure S1 for the experimental setup), and the uncaging was monitored by HPLC (Figure 2). After 2 min irradiation, only trace amounts of caged MSNs remained, and the corresponding free MSNs were formed quantitatively without detectable nucleotide byproducts demonstrating a clean and efficient photocleavage.

### 2.3 Uptake of MSN Probes Into *Escherichia coli*

**FIGURE 3:**
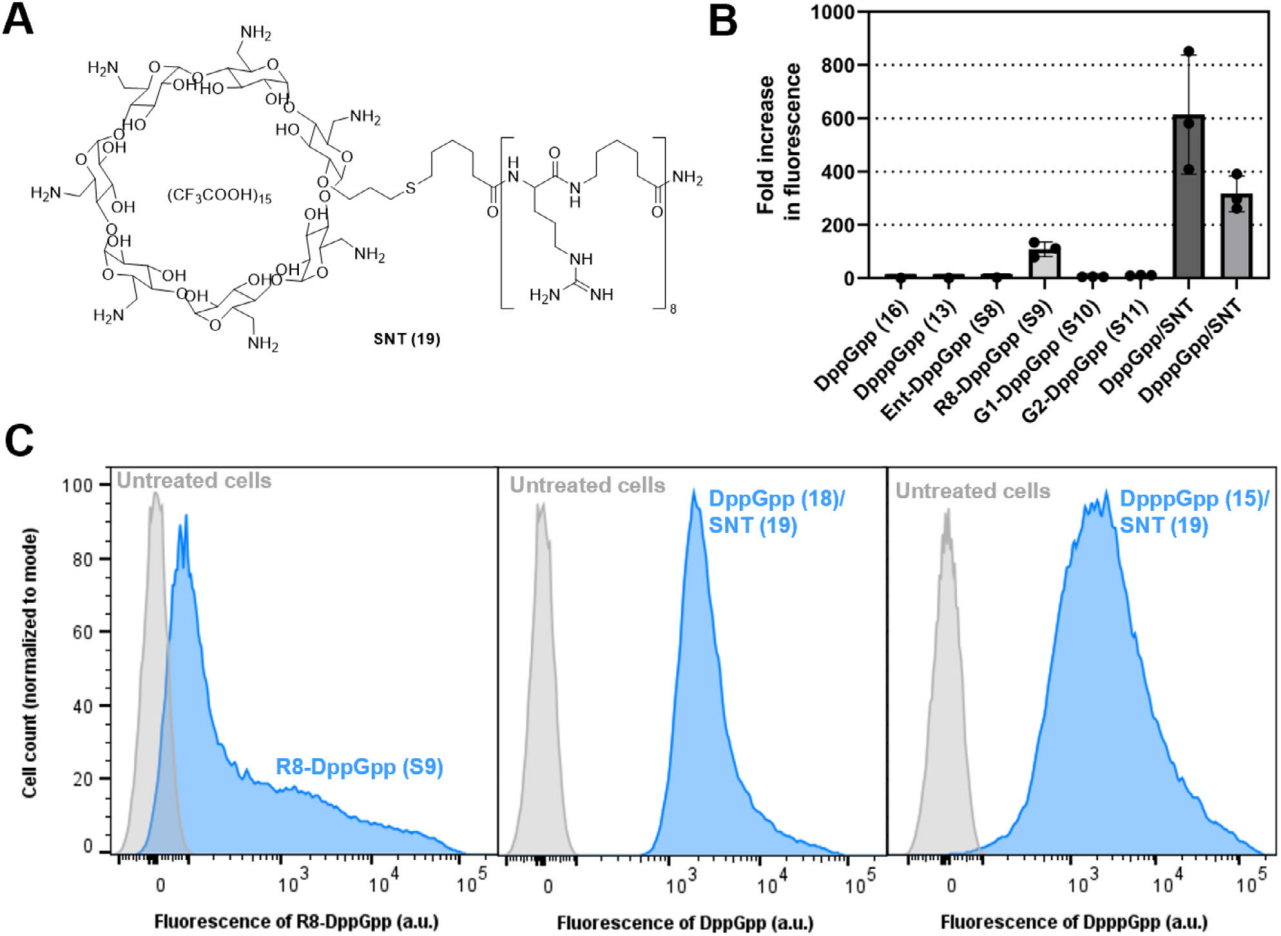
Uptake measurements of MSN in *E. coli*. (A) Structure of the synthetic nucleotide transporter (SNT, 19). (B) Cellular uptake determined by measuring fluorescence of cell suspensions. Bacteria were either treated with the indicated nucleotide alone or with a mixture of SNT (19) and DppGpp (18, DppGpp/SNT) or DpppGpp (15, DpppGpp/SNT). Values were normalized to the autofluorescence of cells treated with DppGpp or DpppGpp alone. Mean ± standard deviation is shown. (C) Flow cytometry histograms of *E. coli* treated with R8‐DppGpp (S9), DppGpp/SNT, or DpppGpp/SNT.

Next, uptake into bacteria was assessed by incubating *E. coli* cell suspensions with solutions of the caged nucleotides. After incubation, the cells were separated by centrifugation, washed, resuspended, and fluorescence of the cell suspension was measured. The intrinsic fluorescence of the DEACBY caging group enabled this rapid initial study of potential delivery. MSN derivatives coupled to carbohydrates and siderophore (**S1‐8**) did not produce fluorescence above the autofluorescence of *E. coli* cells (Figures 3B and S2) under the experimental conditions. The generation 1 and generation 2 PAMAM dendrons coupled to DppGpp (G1‐DppGpp, **S10**; G2‐DppGpp, **S11**) showed a minor fluorescence increase of approximately five‐ and ten‐fold over autofluorescence, respectively. In contrast, octaarginine coupled to DppGpp (R8‐DppGpp, **S9**) produced an approximately 100‐fold fluorescence increase, providing a first indication of cellular delivery. Flow cytometry further revealed uptake in 66 % of bacterial cells (Figure 3C).

Additionally, we studied the synthetic nucleotide transporter (SNT, **19**, Figure 3A) developed by Kraus [34]. This commercially available compound was mixed with DppGpp (**18**, DppGpp/SNT) and DpppGpp (**15**, DpppGpp/SNT) and then incubated with *E. coli* cell suspensions. After washing, the cell suspensions exhibited a substantial fluorescence increase of more than 300‐fold over autofluorescence (Figure 3B). Flow cytometry confirmed these results (Figure 3C), demonstrating largely increased fluorescence of nearly all bacterial cells. Based on these findings, SNT (**19**) was selected for further studies as a transporter for MSNs into *E. coli*. A preliminary experiment using the transport activators pyrenebutyrate and B_12_Br_12_^2−^ did not further increase DpppGpp/SNT uptake (Figures S3) [62, 79].

### 2.4 Confocal Microscopy Analysis of Cellular Distribution

**FIGURE 4:**
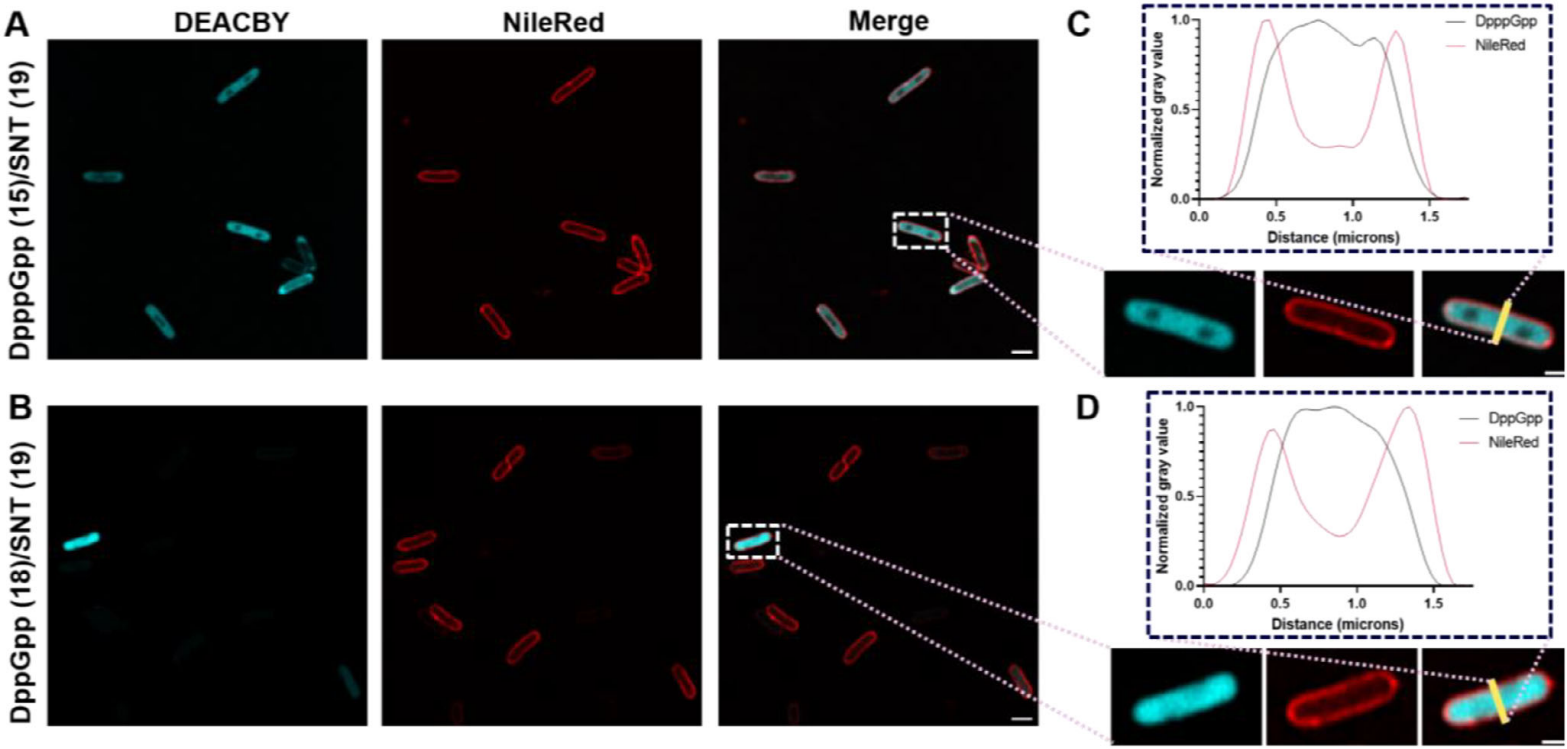
Microscopy images of *E. coli* treated with a mixture of DppGpp (18, B + D) or DpppGpp (15, A + C) with SNT (19) and the membrane stain Nile Red (red fluorescence). The fluorescence of the photolabile protecting group DEACBY is shown in cyan. (C + D) Intensity profiles through a single cell normalized to the highest and lowest grey values. Scale bar = 2 µm in large images and 1 µm in magnified images.

The previously described experiments cannot distinguish whether the MSNs are just bound to the exterior of the *E. coli* membrane or if they are internalized into the periplasm or cytoplasm. Thus, uptake was additionally evaluated with confocal microscopy under optimized conditions using the high throughput methods described above (Figure 4A,B). The cellular membranes were stained with Nile red. The intensity profiles of Nile red fluorescence compared to DEACBY fluorescence show the distribution of the caged MSNs inside the bacterial cells (Figure 4C,D). While Nile red fluorescence is maximal at the start and at the end of the intensity profiles representing the cellular membrane, DEACBY fluorescence is maximal between the Nile red maxima. This clearly demonstrates that the caged MSNs enter the cytoplasm of *E. coli* cells. Additionally, confocal fluorescence images revealed heterogeneous caged MSN uptake into different bacterial cells (Figures S4–S6). Some cells exhibit high intracellular fluorescence, while others show low or even no DEACBY fluorescence, contrasting with the flow cytometry measurements in Figure 3C (see Figure S4: 45% show DEACBY fluorescence).

Cell viability was checked next. Treatment with 50 µL OD_600_^−1^ incubation solution did not affect viability, while increasing the volume to 200 µL OD_600_^−1^ reduced viability to approximately 50% (Figure S7).

### 2.5 Metabolic Tracking of MSNs

**FIGURE 5:**
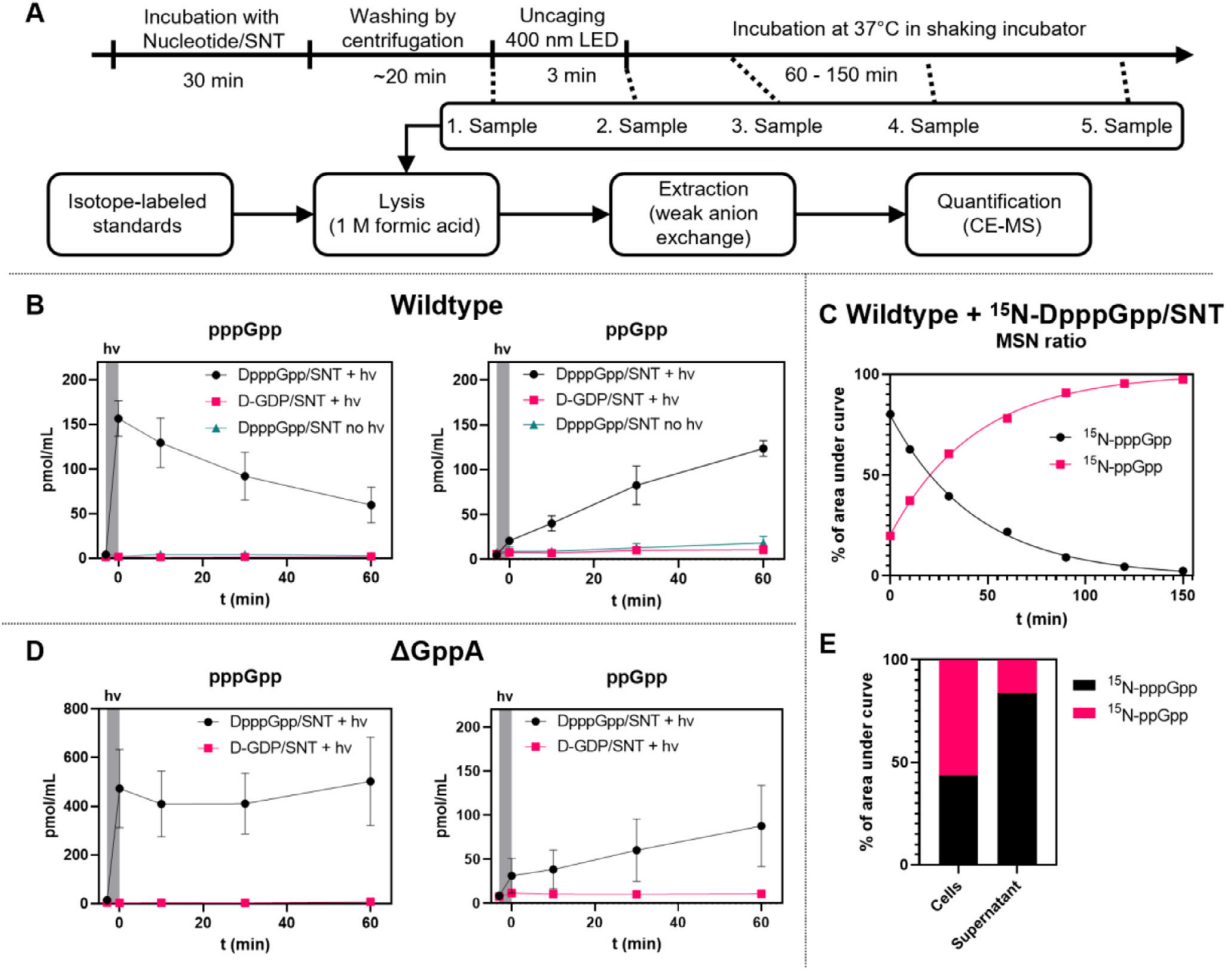
MSNs extracted from *E. coli* after incubation with caged MSNs. (A) Experimental timeline of extraction experiments. (B) pppGpp and ppGpp concentrations extracted from cell suspension of *E. coli* cells incubated with DpppGpp/SNT or D‐GDP/SNT. Cell suspension was irradiated with 400 nm light before *t* = 0 min. (C) Ratio of ^15^N labeled pppGpp and ppGpp extracted from *E. coli* cell suspension incubated with ^15^N‐DpppGpp/SNT and irradiated with 400 nm light. Before irradiation, no ^15^N‐labeled nucleotides were detected. (D) pppGpp and ppGpp concentrations extracted from cell suspension of *E. coli ΔgppA* mutant incubated with DpppGpp/SNT or D‐GDP/SNT. Cell suspension was irradiated with 400 nm light before *t* = 0 min. (E) Ratio of ^15^N labeled pppGpp and ppGpp extracted from *E. coli* cell suspension that was incubated with ^15^N‐DpppGpp/SNT and irradiated with 400 nm light. After uncaging, cells were separated by centrifugation (5 min, 5 kG) and then immediately lysed in 1 M formic acid. Mean and standard error of mean from three experiments are shown in B and D.

In order to confirm that uncaging inside *E. coli* is possible and to track the fate of the released MSNs, we applied an extraction and quantification assay using capillary electrophoresis mass spectrometry (CE‐MS) [73, 80]. After incubation with DpppGpp (**15**) and SNT (**19**) and successive washing, the *E. coli* cells were resuspended in medium. The first sample for MSN extraction was taken, and the cell suspension was irradiated with a 400 nm LED (14 mW, 3 min) to release the free nucleotides (Figure 5A). The culture was then incubated at 37°C for 1 h and samples were collected at predetermined time points before and after irradiation. The samples were lysed, spiked with heavy isotope‐labeled standards and extracted using weak anion exchange. Quantification was subsequently performed by CE‐MS.

After irradiation with 400 nm light, we observed a direct and sharp increase in pppGpp levels, confirming successful photolysis (Figure 5B). Over time, pppGpp levels decreased while ppGpp increased, consistent with phosphatase‐mediated hydrolysis. In *E. coli*, GppA is the phosphatase responsible for the conversion of pppGpp to ppGpp. In fact, ppGpp is the main alarmone in *E. coli* due to GppA activity [81, 82]. This was confirmed using ^15^N‐DpppGpp (16): we detected a corresponding increase in [^15^N]_5_‐ppGpp as [^15^N]_5_‐pppGpp declined (Figure 5C), indicating that the ppGpp originates mostly from the uncaged ^15^N‐labeled pppGpp and not through indirect effects of the uncaging process. Bacteria treated with caged GDP or bacteria that were not irradiated with 400 nm light did not show this change in MSN levels (Figure 5B).

To further validate this assumption, we also performed the extraction experiment with a *ΔGppA* mutant (Figure 5D) [83]. In this strain, pppGpp levels remained stable due to the absence of GppA; however, ppGpp still accumulated, consistent with previous reports that pppGpp allosterically activates RelA, thereby enhancing ppGpp synthesis [12, 15].

We performed another control: Bacteria were treated with DpppGpp (**15**) and SNT (**19**) and [^15^N]_5_‐pppGpp was spiked into the medium directly after uncaging. The conversion of the heavy‐isotope labeled [^15^N]_5_‐pppGpp to [^15^N]_5_‐ppGpp resembled the conversion of pppGpp to ppGpp (Figure S8), even though [^15^N]_5_‐pppGpp should only be present in the medium, while pppGpp should be located inside the bacterial cells. These results could imply that periplasmic or extracellular phosphatases, such as PhoA [84] and/or phosphatases released from damaged cells can also process MSNs. Furthermore, damaged cells may release MSNs into the culture medium or take up ^15^N‐labelled MSNs from the medium.

To better understand which portion of the quantified nucleotides were truly intracellular, we separated the cell pellets and the culture supernatants after photolysis by centrifugation and analyzed both fractions. We could detect MSNs in both fractions, but surprisingly, the supernatant contained more pppGpp but less ppGpp than the pellet fraction (Figure S9). This shows that MSNs leak into the medium, potentially through the action of the SNT. However, rapid intracellular metabolism could reduce detectable MSN levels inside the cells, exaggerating the relative abundance measured in the supernatant. When we repeated these experiments with ^15^N‐DpppGpp (**16**), we found that pppGpp‐to‐ppGpp conversion was indeed markedly faster in the pellet fraction, whereas extracellular conversion was slower (Figure 5E).

Next, we investigated whether a drug efflux system could be responsible for MSN leakage into the medium [85, 86, 87]. Therefore, we compared knockout strains lacking the major drug efflux components AcrA, AcrB, and TolC with the wild type [83]. In a simple experiment, we incubated the cell suspensions with DpppGpp/SNT and performed four washing steps where we centrifuged the cell suspension, removed the supernatant and resuspended the pellet. We then analyzed the washing solution for DEACBY fluorescence to measure DpppGpp leakage. Even after four washing steps, significant amounts of DpppGpp could still be detected in the washing solution of all the strains investigated (Figure S10). This finding suggests that SNT‐induced membrane perturbation, as opposed to active export of internalized caged MSN, serves as the predominant contributor to the observed phenomenon.

Taken together, these data suggest that the extracellular MSN pool arises largely from membrane damage, which releases (p)ppGpp into the medium. Concurrently, rapid intracellular turnover depletes the internal MSN pool, resulting in lower intracellular than extracellular levels. Thus, although CE‐MS provides robust tracking of MSN photo‐release and metabolism, careful interpretation is required as it may partially reflect extracellular rather than intracellular pools. Notwithstanding, confocal microscopy unambiguously showed internalization of the probes into a fraction of cells. We, therefore, conclude that while a fraction of the probe is released intracellularly, another fraction is leaking from cells as a consequence of SNT‐mediated membrane damage.

### 2.6 Bacterial Growth Assay

The bacterial growth rate is apparently regulated by MSN [88, 89, 90]. As simple read‐out we envisioned that photo‐release of caged MSN should slow down bacterial growth. Therefore, the growth of *E. coli* was monitored after incubation with the D(p)ppGpp/SNT mixture with and without following photo‐release.

For wild type *E. coli*, we could not detect this projected difference in growth rate. As shown in the confocal microscopy experiments described above, uptake of the caged MSNs was heterogeneous. We, therefore, attribute the absence of a measurable population‐level effect to this cell‐to‐cell variability: while some cells internalized high amounts of D(p)ppGpp, others likely contained only minimal levels, resulting in no apparent change in overall growth.

**FIGURE 6:**
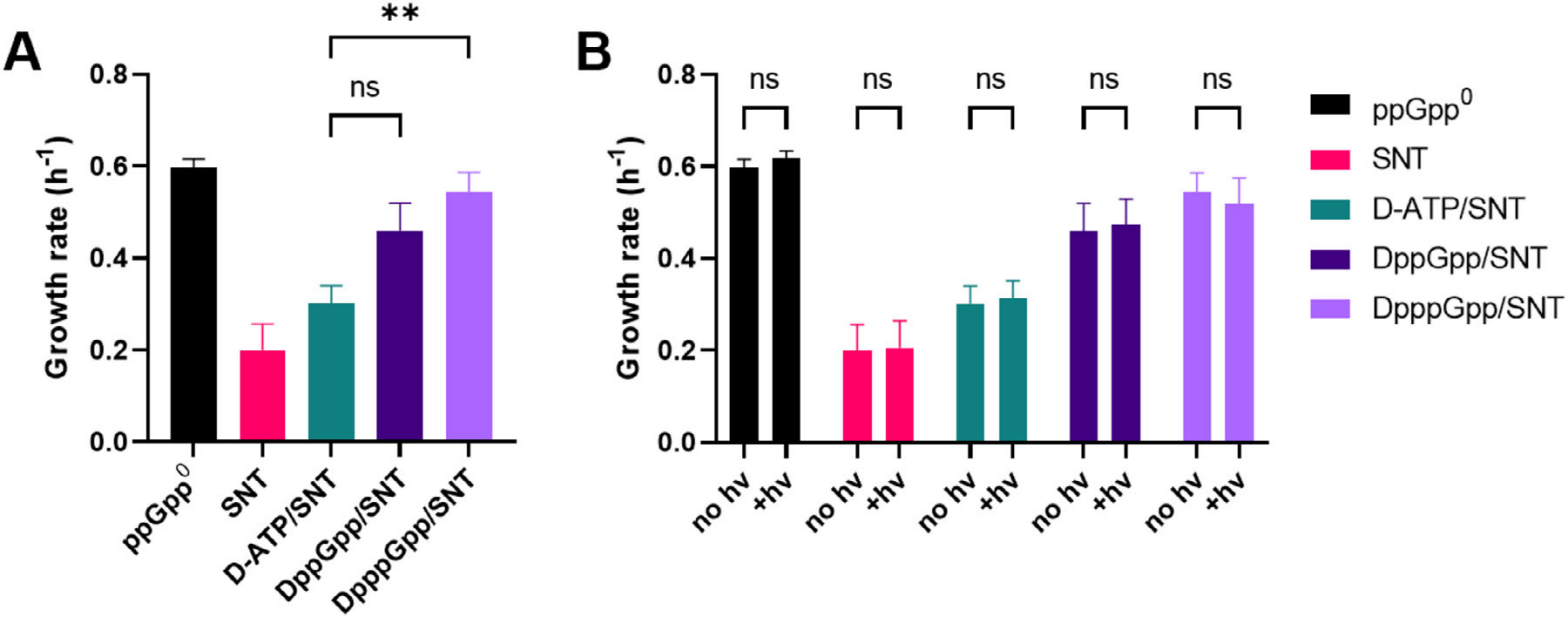
Growth rate of the *E. coli* (p)ppGpp^0^ mutant treated with SNT and different nucleotides. (A) Comparison of the different treatment conditions without light irradiation. Analyzed by Brown–Forsythe and Welch ANOVA test with Dunnett′s T3 multiple comparisons test; individual variances computed for each comparison. ns: *p* = 0.125; **: *p* = 0.0054. (B) Comparison of samples irradiated or not irradiated with 400 nm light. Analyzed with two‐way ANOVA with Šídák multiple comparisons test, with a single pooled variance. ns: *p* > 0.99. Mean and standard error of mean of at least four separate experiments is shown.

To overcome this limitation, we switched to a mutant strain incapable of synthesizing MSNs ((p)ppGpp^0^) due to deletion of the *relA* and *spoT* genes. As basal levels of MSNs are important to maintain cellular component homeostasis, this mutant has multiple disabilities in cell division, transcription, and translation and can have difficulties to adapt to environmental changes [4, 6]. Due to the absence of MSNs in this mutant, the influence of artificially added MSNs on the growth rate should be pronounced, but in this case, potentially increase the growth rate due to compensation of above‐mentioned defects. We observed that treatment with SNT alone decreases the bacterial growth rate, likely due to its toxicity by damaging the bacterial envelope. To evaluate the influence of DppGpp (**18**) and DpppGpp (**15**) mixed with SNT (**19**) on the growth rate of the *E. coli* (p)ppGpp^0^ strain, we used D‐ATP (**S12**) mixed with SNT (**19**) as a control. Interestingly, incubation of (p)ppGpp^0^ with SNT (**19**) mixed with caged (p)ppGpp (**15**, **18**) already recovered the loss in growth rate caused by SNT. Caged ATP (**S12**) did not show this effect (Figure 6A). One possible conclusion is that caged MSNs, due to their versatile binding patterns [91], can already interact with some of the binding partners of MSN and thus restore growth. Supporting this interpretation, uncaging of (p)ppGpp did not further affect the recovery of the growth rate (Figure 6B). This interpretation is in line with previous findings for caged molecules [66, 92, 93]. Future studies will now address the increase of bulkiness in the cage through click chemistry on the alkyne handle to abrogate unwanted binding prior to uncaging [94].

## 3 Summary and Conclusion

In this study, we present the first photocaged derivatives of the MSNs pppGpp and ppGpp. DppGpp (**18**), DpppGpp (**15**), and the heavy isotope labeled ^15^N‐DpppGpp (**16**) were efficiently synthesized in high yields using a chemoenzymatic synthesis approach. By comparing different delivery strategies, we achieve the delivery of highly negatively charged, caged MSNs through the complex cell envelope of living *E. coli*. Several analytical approaches combined provide evidence for intracellular delivery into a subpopulation of cells. Upon 400 nm irradiation, these probes release free nucleotides, enabling tracking of pppGpp‐to‐ppGpp conversion by CE‐MS. Moreover, initial growth curve studies in wild type and (p)ppGpp^0^ strains demonstrate the potential of these probes for in vivo studies after further optimization. While photolysis and metabolic conversion were clearly demonstrated, extraction experiments revealed substantial leakage of MSNs into the extracellular medium, complicating accurate quantification of intracellular levels. Growth studies with a (p)ppGpp^0^ strain confirmed that caged MSNs can modulate bacterial physiology, but no light‐dependent growth effect was yet observed, likely due to residual activity of the caged species.

Future work should prioritize improved transporter strategies and the development of bulkier, biologically inert cages. Single‐cell studies of cells that have received large amounts of caged nucleotide could help attribute heterogeneous uptake to specific cellular states. Despite its current limitations, this study provides first significant strategies how to deliver highly charged signaling molecules into living bacteria and control cellular concentrations on a minute time‐scale through photo‐uncaging.

## Author Contributions

**Christoph Popp**: synthesis, investigation, conceptualization, formal analysis, visualization, writing. **Patrick Moser**: synthesis of ^15^N‐DpppGpp (16). **Anselm Schwoerbel**: investigation. **Xinwei Liu**: supervision and discussion of in vivo experiments. **Johannes Freitag**: supervision and discussion of in vivo experiments. **Pinku Sarmah**: supervision and discussion of uptake experiments. **Isabel Prucker**: discussion of CE‐MS results. **Robert Zscherp**: synthesis of S11. **Xuan Wang**: synthesis of S13. **Philipp Klahn**: supervision and discussion. **Hans‐Georg Koch**: supervision and discussion. **Gert Bange**: supervision and discussion of in vivo experiments. **Henning J. Jessen**: conceptualization, supervision, funding acquisition, writing – review and editing.

## Conflicts of Interest

The authors declare no conflicts of interest.

## Supporting information




**Supporting File 1**: anie71610‐sup‐0001‐SuppMat.pdf.

**Supporting File 2**: anie71610‐sup‐0002‐Data.xlsx.

## Data Availability

The data that support the findings of this study are available in the supporting information of this article.
